# International Summit 2014: Organisation of clinical ultrasound in the world

**DOI:** 10.1007/s13244-014-0358-9

**Published:** 2014-11-06

**Authors:** European Society of Radiology (ESR)

**Affiliations:** Neutorgasse 9/2, 1010 Vienna, Austria

## Abstract

Ultrasound (US) is a widely used imaging modality throughout the world, yet differences in usage remain among countries or regions, according to the results of the International Summit, organised by the ESR during the European Congress of Radiology last March in Vienna. The International Summit is held each year by the ESR and its partner national and international societies of radiology from outside Europe with the primary goal of gathering information about a particular topic in radiology from a worldwide perspective. In 2014, some aspects of the practice of US imaging within and outside radiology were discussed, following a list of items prepared by the ESR Working Group on Ultrasound. Results showed that radiological US has similar problems throughout the world. At the same time, however, there are profound differences in how US is practised and the results of this meeting should be considered with caution. The results of the International Summit offer an overview of the major trends and differences in the use of US worldwide, but as a whole suggest that this imaging technique still plays a major role in radiology and health care.

Main messages

• US is a widely used modality and constitutes a great part of radiological workload.

• The use of ultrasound is split between radiological and non-radiological services.

• Training differs among countries and the presence of local subspecialty societies improves training quality.

• The shortage of local radiologists and lack of interest among young radiologists are worrying.

• US use should not be limited to radiologists alone, especially in sparsely populated areas.

The International Summit is a closed meeting organised by the ESR to address and discuss problems of global interest with its partner national and international societies of radiology from outside Europe. It is held each year during the European Congress of Radiology and its main aim is to gather information about a particular topic in radiology from a worldwide perspective. The meeting is chaired by the ESR President, and the topic is chosen each year by the ESR Communication and External Affairs Committee Chairperson.

At ECR 2014, 11 national and international societies presented and discussed some aspects of the practice of US imaging within and outside radiology during this special meeting.

Each society answered a list of items prepared by the ESR Working Group on Ultrasound. These followed the questions prepared for the survey conducted in 2012 among radiological departments in Europe, the results of which were published in this journal in August 2013 [1].

The answers showed that radiological US has problems that are quite similar throughout the world. At the same time, however, there are profound differences in how US is practised. Therefore, the results of the meeting need to be considered with caution. One of the main problems was that the response rate was rather low and unevenly distributed around the world. For instance in Europe, only 123 heads of radiology departments answered the questionnaire while 64 affiliated hospitals conducted the survey in Japan. Only 12 of the 22 contacted members of the Asian Oceanian Society of Radiology (AOSR) took part in the survey, making it challenging to summarise the status of ultrasound in this region, which presents varied scenarios depending on the country and institution. Last but not least, many developing countries still have little or no access at all to US imaging.

The results thus offer an overview of the major trends and differences in the use of ultrasound around the world, but as a whole suggest that ultrasound still plays a major role in radiology—and sometimes—other departments.

## What are the fields of application of radiological US?

Surveys unanimously reveal that US studies of the abdomen in adults are the most common investigation performed within radiology throughout the world. In the USA alone, almost 4 million abdominal US examinations were performed in 2012, accounting for 57.2 % of all US examinations performed by radiologists in the country. Similarly, abdominal US examinations represent 49 % of all radiological investigations in South Korea, and 47 % in Japan, 40 % in India, 38.5 % in Latin America and 32 % in Colombia.

Breast US remains a very frequently performed examination worldwide; it is the second most common US study in Latin America. Foetal examinations represent 40 % of all US investigations in India and are also frequent in Colombia. Paediatric examinations are the second most common studies performed by radiologists in the USA; outside of radiology, cardiac examinations are by far the most common US investigations in this country.

In Korea, examinations of the female pelvis are the most common studies after abdominal imaging, ahead of MSK and vascular examinations. MSK investigations are carried out frequently in Australia and in Japan, where they represent, together with foetal, the most common US studies performed by both radiologists and non-radiologists.

## How is US used by other medical specialists?

The use of ultrasound is split between radiological and non-radiological services, and ultrasound machines are scattered throughout departments in almost all hospitals. A few hospitals have a centralised ultrasound laboratory where all specialists perform examinations, each according to his/her clinical or radiological expertise. This is probably the way forward in optimising resources, with US scanners operating for longer hours and with higher numbers of examinations. However, many physicians prefer to remain independent, and integrating activities is difficult.

In Europe, about 38.32 % of all US studies are carried out by non-radiologists. The most frequent examinations performed outside of radiology are carried out by obstetrics/gynaecology, neurology, vascular surgery, urology, gastroenterology and internal medicine specialists. In addition, new specialities, such as anaesthesiology and emergency medicine, are pushing strongly for US to be available as a diagnostic tool in their fields.

In Latin America, reliable data regarding the type and percentage of US examinations performed by non-radiologists are not available. However, in general terms, pelvic, obstetric and foetal studies are the most frequent examinations, followed by vascular and breast studies. In Colombia, radiologists perform 70 % of all US examinations and most abdominal studies. Breast US is not performed at the same time as mammography unless indicated by the radiologist. Other specialists may use US only within their own areas of medical expertise.

The situation varies widely in Asia. In Japan, there are more than 100,000 US machines in clinical use and most US examinations (88 %) are performed by non-radiologists. In Korea, radiologists perform most US studies.

## What is the role of sonographers?

In the USA, sonographers carry out the studies and radiologists render interpretations, regardless of the type of study performed. The role of sonographers is to perform the initial scan, which is reviewed by a radiologist who confirms the findings and dictates the report. Scans performed off site are reviewed in real time and additional views are obtained if needed. The radiologist reviews the study and dictates the report.

In South and Central America, it is not very common for sonographers to perform US studies themselves except in teleradiology (see below). Their contribution is usually around 10 % of all US studies, except in Puerto Rico (95 %) and Panama (30–70 %). Radiologists then write the report. Usually, sonographers do not perform studies in ultrasound laboratories run by radiologists and do not report US studies; nevertheless, sonographers and nurses perform US studies in vascular ultrasound laboratories.

Sonographers report in China and most of the Japanese institutions, whereas they only carry out the examination in other Asian-Oceanic countries. In India, only qualified postgraduate doctors and radiologists are allowed to perform US examinations. Residents under supervision of senior lecturers do all routine and emergency US. In Korea, sonographers are responsible for the image acquisition, and radiologists and clinicians for the interpretation.

In Australia, the vast majority of ultrasound examinations are performed by sonographers, who undergo 4 years of practical and academic training. They then work as registered sonographers under the guidance of radiologists.

## How is training in radiological US organised?

European radiology residents work under the direct supervision of senior staff, enabling the progressive acquisition of skills, from scanning and reporting capabilities to complete independence. Training is organised mostly according to organ systems after an initial period of technique-oriented teaching. To help support training, there are sections in many national radiological societies dealing with radiological US. In addition, radiologists are quite often involved in the national and European societies specifically dedicated to US.

In the USA, US is part of the 4-year radiology residency programme, and it is up to each programme to integrate ultrasound teaching into the curriculum. There is no minimum requirement of time spent studying ultrasound, but the Accreditation Council for Graduate Medical Education (ACGME) requires at least 340 examinations. Most radiology residents perform many more US examinations during residency. Residents at the University of Michigan, for example, perform on average 640 examinations.

The American Board of Radiology incorporates US and US physics into its testing. As of July 2013, 4,183 ultrasound facilities were accredited by the ACR and 700 facilities were in the process of accreditation. The Society of Radiologists in Ultrasound and the American Institute of Ultrasound in Medicine further promote the education of radiologists in this field.

The situation is alarming in Latin America, according to CIR President Prof. Gloria Soto Giordani. “Training is difficult due to the heavy US workload and shortage of radiologists. The practice of US by non-radiologists, with the exception of cardiac and foetal-obstetrics, is neither officially recognised nor accepted by radiologists as a legitimate practice in most Latin American countries, and there is hardly any coordination between radiologists and non-radiologists performing US,” she said.

In Colombia, the minimum length of training in US for radiology residents is 24 weeks. There is no minimum requirement for ultrasound studies performed. Currently, there is no specific group or society for radiological ultrasound in the country.

The average training period for Japanese residents is 2.4 months but as many as 27 % of the residents have no involvement in US. “Residents need to learn and experience diagnostic radiology, as well as ultrasound, considering the increasing importance of fusion imaging such as US-CT," said Japan Radiological Society President, Prof. Hiroshi Honda. The Japanese Society of Ultrasonics in Medicine (JSUM) is an independent society promoting the use of the modality in Japan.

In India, a minimum of 12 months in US is required with no specific number of examinations during postgraduate training.

In Korea, there is no obligatory time or quantity of examinations to be performed. The Korean Society of Ultrasound in Medicine organises dedicated educational seminars and the Korean Society of Radiology also includes educational events. The KRS underlined the existence of turf battles between clinicians regarding US.

In all the responding societies in Asian-Oceanic countries, ultrasound is included as a part of basic radiology training. Only 4 of 12 responders reported having specific societies for radiological ultrasound.

## What is the situation in developing and low-population density countries?

Remote interpretation of US studies is a fundamental aspect of clinical practice in many regions, especially areas with a low population density such as Australia or Latin America.

The shortage of local radiologists is a topic of concern in the latter, the survey suggests. “Sonographers perform the studies and send the images for telereporting of US exams in the absence of local radiologists, and this is becoming an issue of increasing concern,” Prof. Soto Giordani said. There is a lack of interest among young radiologists in US as it is more time-consuming and financially less attractive than other modalities.

In Australia, tele-US is practiced widely. Many patients are up to 400 km away from an area where they can be examined, whereas teleradiology is available within their vicinity. In practices serving remote communities, more than 50 % of performed examinations are reported through tele-ultrasound.

All US teleradiology examinations have to be performed by sonographers according to a standard procedure, according to Prof. Jan Labuscagne, President of the International Society of Radiology. He stressed that many developing countries have little or no access at all to US and that increasing access to imaging is vital. “Therefore, performing US by non-radiologists should be supported. This should not be seen as competition, since there is currently nobody doing it in these areas,” he said.

This issue is handled by the World Federation of Ultrasound in Medicine and Biology (WFUMB), a multidisciplinary international organisation in the field of ultrasound. The WFUMB resolution says that the practice of investigative ultrasound is open to a wide variety of medical specialties of which none has any exclusive right to work in a particular area. “The primary criteria that should be considered, in any situation where it is necessary to assign to particular specialists responsibility for practice of investigative ultrasound, are the medical well-being of patients and cost effectiveness,” said WFUMB Education Committee Chairperson, Prof. Byung Ihn Choi.

He emphasised that radiologists should not abandon US but keep their position as imaging specialists and supervisors of education, research and clinical practice of medical US for the sake of medical well-being, the best care for patients and optimal cost-effectiveness, and not for their own financial benefit.

## Ongoing discussion

There is a shortage of radiologists for US examinations in the developing world and WFUMB is trying to promote tele-interpretation of US studies in these countries. For example, there is a shortage of radiologists in Africa, and US could be performed for many basic examinations in these areas. Therefore, midwives and nurses are being taught to carry out US examinations and radiologists should be there as supervisors, as due to the shortage, they do not have the time to perform standard examinations.

Furthermore, it has been suggested that in these regions students should already be taught to perform first level and minor procedure ultrasound examinations in medical schools where radiologists and experts can supervise. Experts underlined that the majority of the radiologists’ income comes from ultrasound and compared the economic situation to the main objective, which is to provide quality care, no matter who is reporting, in developed and developing countries. They insisted the focus should be on the clinical application. Efforts should be aimed at providing adequate training to those performing the US studies and standardising the examinations so that quality standards can be reached everywhere.

Experts agreed that there is no one solution for the whole world and that quality work in US can also be done by non-radiologists if the circumstances require it in certain settings and if appropriate training is provided.
